# Using High-Resolution Differential Cell Counts (HRDCCs) in Bovine Milk and Blood to Monitor the Immune Status over the Entire Lactation Period

**DOI:** 10.3390/ani12111339

**Published:** 2022-05-24

**Authors:** Sabine Farschtschi, Alex Hildebrandt, Martin Mattes, Benedikt Kirchner, Michael W. Pfaffl

**Affiliations:** Division of Animal Physiology and Immunology, TUM School of Life Sciences, Technical University of Munich, 85354 Freising, Germany; alex.hildebrandt@tum.de (A.H.); martin.mattes90@web.de (M.M.); bkirchner@tum.de (B.K.); michael.pfaffl@tum.de (M.W.P.)

**Keywords:** differential cell count, differential leukocyte count, somatic cell count, dairy cow, immunomonitoring, immunophenotyping, udder health

## Abstract

**Simple Summary:**

Since leukocytes enter the mammary gland as part of the immune defense, they can be analyzed in raw milk samples and used for diagnostics. In our study, we differentiated and measured several subtypes of immune cells in blood and milk samples of eight dairy cows over their whole lactation to monitor their immune status. In addition to these so-called high-resolution differential cell counts (HRDCCs), we determined routine laboratory biomarkers. We found considerable differences between the documented cell count progressions in the blood and milk. Some cell populations remained mostly stable, while others showed a certain dynamic during the course of lactation. Moreover, two types of lymphocytes were also noticeable due to changes in the case of inflammation. These findings suggest that HRDCCs can be a promising tool for more efficient milk diagnostics, ensuring milk quality, and supporting cattle health and wellbeing.

**Abstract:**

Differential cell counts in milk offer a deeper insight into the immunology of the mammary gland and might even provide information about the systemic health status of a dairy cow. Consequently, their potential as a diagnostic method to identify biomarkers has been a subject of research for quite some time. The objective of our study was to closely monitor the immune status of eight healthy dairy cows throughout their whole lactation. For this, high-resolution differential cell counts in milk and blood were determined by means of flow cytometry, which included 10 subpopulations of the 3 main populations of immune cells and their viability. Milk and blood samples were taken twice a week in the first 100 days after calving and once a week during the remaining lactation period: in total, 55 (52–57) blood and 55 (52–57) milk samples per animal. In addition, six well-established routine laboratory biomarkers, i.e., haptoglobin, calcium, and different metabolic parameters (non-esterified fatty acids, β-hydroxybutyric acid, bilirubin, and glutamate dehydrogenase), were analyzed in all blood samples. Furthermore, a standard differential blood cell count was performed on all blood samples. We found substantial differences between cell count progressions in the blood and milk. The distribution of cell populations in the blood remained mostly stable throughout the lactation, albeit at different individual levels. Several cell populations in the milk showed a noticeable dynamic over time, which caused a separation of different lactation stages in clustering analyses. Gamma delta T cells and CD4^+^ T cells in the milk stood out as they showed characteristic fluctuations during the course of lactation as well as minor changes in the case of inflammation. The determination of a differential cell count has the potential to be a sensitive diagnostic and prognostic tool in bovine milk. Further studies need to show to what extent the method is suitable for routine diagnostics and how to deal with animal-specific differences.

## 1. Introduction

In recent decades, structures in dairy farming have changed fundamentally in industrial countries, as extensively reviewed by Barkema et al. [1]. These transformations lead to a reduction in the number of farms and, at the same time, to an increase in herd sizes. Especially on larger dairy farms, where it can be more challenging to detect diseased cows, health monitoring strategies and efficient biomarkers are needed to control animal health and meet consumer demands for food safety and animal welfare [2,3]. Bovine diseases—first and foremost mastitis—not only impair the wellbeing of dairy cows but are also accompanied by major financial losses [4,5]. Therefore, it is beneficial to diagnose diseases at an early stage, as shown, for example, by van den Borne et al. [6] for subclinical intramammary infections. Moreover, the high consumption of antibiotics used for therapy and prophylaxis on dairy farms [7] can lead to an increase in antibiotic-resistant bacteria, which pose a danger to both humans and animals [8]. In this respect, an early diagnosis and thereby a reduced spread of infection among herd mates are crucial.

As a monitoring tool for mastitis, the determination of a somatic cell count (SCC) has been well established in routine milk diagnostics since the 1950s [9], either for an individual cow or at the herd level. The SCC is, for example, a standard parameter in Dairy Herd Improvement (DHI) testing [10] and can currently be included in automatic milking systems [11]. However, the informative value of the SCC is limited by the fact that it can be affected by other confounding factors besides intramammary infection, e.g., by breed, parity, or lactation stage [12,13]. To further advance milk diagnostics, we can take advantage of the fact that milk comprises various populations of immune cells, which have different biological functions [14,15] and thus offer additional information. The determination of a differential cell count (DCC) has been suggested to provide an expanded information base about intramammary infections and the health state of a mammary gland [16,17,18], or even about the systemic health condition [19]. Schwarz and colleagues [20] defined different udder health groups based on a DCC that represents the combined proportion of polymorphonuclear neutrophils and lymphocytes as a percentage of the total SCC. Cows were categorized into these four groups according to their SCC and DCC results of the last DHI tests.

With this long-term study, we present the results of our high-resolution differential cell count (HRDCC) method in milk and blood that were acquired by means of flow cytometric measurements over the entire lactation period. These HRDCC numbers document the spectrum in which the physiological counts range, and cow individual differences.

## 2. Methods

### 2.1. Animal Study

Eight Brown Swiss cows between the first and the fifth lactation were included in this study and sampled throughout their entire lactation, i.e., on average, 300 (291–305) days. The cows were kept under German standard conditions in compliance with good agricultural practice. They were housed at Veitshof, a research station of the TUM School of Life Sciences (Technical University of Munich in Freising, Germany), in a cubicle housing system with rubber-coated slatted floors. Milking took place twice a day in a 2 × 2 tandem milking parlor (GEA WestfaliaSurge GmbH, Bönen, Germany). Cows had ad libitum access to fresh water, and the daily feed ration consisted of 18 kg corn silage, 14 kg grass silage, and 1.5 kg hay on average, supplemented with 1.5 kg high-protein rape and soy extraction meal (deuka Kompopur 404, Deutsche Tiernahrung Cremer, Düsseldorf, Germay) and 190 g mineral mix (Complett Keragen Longlife, Josera, Kleinheubach, Germany). In addition, 0.5 kg concentrated feed with 19% crude protein (deuka MK 194-UDP, Deutsche Tiernahrung Cremer, Düsseldorf, Germany) per liter of delivered milk was added to the diet to assure the necessary energy intake. This study was conducted in concordance with the German Animal Welfare Act (TierSchG) and the German regulations on the welfare of animals used for experiments or for other scientific purposes (Tierschutz-Versuchstierverordnung, TierSchVersV). The government of Upper Bavaria in Munich, Germany, permitted the animal study (reference number ROB-55.2-2532.Vet_03-17-70). All cows included in this study were examined by a veterinarian at least on each day of sampling, and their physical condition was documented.

### 2.2. Sampling, Cell Isolation, and Staining

Blood and milk samples were collected twice a week for the first 100 days in milk (DIM) and once a week for the rest of the lactation. On average, 55 (52–57) blood and 55 (52–57) milk samples were taken from each animal. The sampling procedure and sample preparation were extensively described by Farschtschi et al. [19]. In short, milk samples were obtained during the whole morning milking process in the milking machine and brought directly to the laboratory without any added preservative. They were centrifuged three times, and cell pellets were washed with DPBS (Dulbecco’s Phosphate Buffered Saline, Sigma Aldrich, Co., St. Luis, MO, USA). Blood was sampled from the jugular vein after morning milking, incubated with a lysis buffer, and centrifuged three times. Afterwards, isolated cells were counted with a TC10 Automated Cell Counter (Bio-Rad Laboratories Inc., Hercules, CA, USA). Separate samples with 10^6^ milk or blood cells were stained on ice with a viability dye (Zombie NIR Fixable Viability Kit, Biolegend, Inc., San Diego, CA, USA) and different antibody mastermixes (Tables S1–S3) and fixated for subsequent analysis on the next day. Appropriate FMO (fluorescence minus one) controls were prepared using isotype control antibodies (Table S4).

### 2.3. Flow Cytometric Analysis and Gating

All samples were analyzed with a BD LSRFortessa flow cytometer (Becton, Dickinson and Company, Franklin Lakes, NJ, USA) with four lasers and the appropriate BD FACSDiva software. Prior to every experiment, AbC Total Antibody Compensation Bead Kit (Thermo Fisher Scientific Inc., Waltham, MA, USA) was used to compensate for any overlap of emission spectra of the fluorochromes, if necessary. Raw data were assessed with FlowJo software 10.7.1 (Becton, Dickinson and Company, Franklin Lakes, NJ, USA). In addition, the automated gating tool openCyto [21] was applied to improve the reproducibility of the results. This R package allows setting gates automatically according to the pattern of the manual gating. Both results were compared using the R package flowWorkspace [22] to evaluate the agreement between the two gating methods. We already described the gating strategy for our HRDCC in detail [19], and an exemplary gating can be seen in the Supplementary Materials (Figure S1). In brief, in the first steps, most of the debris as well as doublets and dead cells was excluded. Afterwards, CD45^+^ cells, i.e., leukocytes [23], were distinguished into granulocytes, macrophages/monocytes, or lymphocytes based on their granularity, which could be ascertained by their SSC values. We differentiated eosinophils from the rest of the granulocytes (CD45^+^SSC^high^) based on their autofluorescence [24]. Immature granulocytes were identified by their lower expression of CD11b [25]. The monocytes (CD45^+^SSC^mid^) in the blood were separated into their subsets [26] of classical monocytes (cM, CD14^+^CD16^−^), intermediate monocytes (intM, CD14^+^CD16^+^), and nonclassical monocytes (ncM, CD14^−^CD16^+^). We took a similar approach with the macrophages (CD45^+^SSC^mid^) in the milk by subdividing them into classical macrophages (cMac, CD14^+^CD16^−^) and nonclassical macrophages (ncMac, CD14^+/−^CD16^+^). Furthermore, the following subpopulations of lymphocytes (CD45^+^SSC^low^) were analyzed: natural killer (NK) cells (CD335^+^), gamma delta T cells (gdTCR^+^), CD4^+^ T cells, CD8^+^ T cells, and B cells (CD21^+^). In addition, we used a pan cytokeratin marker to determine the amount of mammary epithelial cells (MECs) in CD45^-^ milk cells. Due to problems with the respective antibody, MECs could only be recorded starting from 50 DIM.

### 2.4. External Analysis of Blood Samples

An additional 55 (52–57) EDTA blood and serum samples were analyzed at a veterinary laboratory (Laboklin GmbH & Co. KG, Bad Kissingen, Germany) for routine biomarkers and a differential blood count. The levels of the acute phase protein haptoglobin, calcium and metabolic parameters, i.e., non-esterified fatty acids (NEFA), β-hydroxybutyric acid (β-HBA), bilirubin, and glutamate dehydrogenase (GLDH), were determined by means of multiple photometric assays with the Cobas c701 system (F. Hoffmann-La Roche Ltd., Basel, Switzerland). Moreover, a standard differential blood cell count was performed on all blood samples using ADVIA 2120i (Siemens Healthcare GmbH, Erlangen, Germany).

## 3. Statistical Analysis

Prism 9.3.1 (GraphPad Software Inc, San Diego, CA, USA) was applied to examine, assess, and visualize the data, especially the progression of cell counts over time, using, e.g., third-order polynomial interpolation (outliers eliminated, Q = 1%), linear regression, and smoothing splines (using four knots). In addition, we used multivariate statistical analyses, herein unsupervised and supervised clustering techniques, to evaluate the comprehensive amounts of data and investigate changes in all cell counts (except MECs) during the course of lactation. We therefore divided the lactation period into different phases with 50 DIM each to see which parameters vary the most between them. First, an unsupervised principal component analysis (PCA) was performed with Base R [27] and the R package Factoextra [28], followed by a supervised sparse partial least squares-discriminant analysis (sPLS-DA) using the R package mixOmics [29].

## 4. Results

### 4.1. Comparison to Automated Gating

More than 11,500 manually set gates were compared to those automatically created. The simple linear regression analysis of both gating strategy data sets showed an overall significant agreement and hence the validity of the user-independent and automatic gating strategy (*n* = 11,574, r = 0.9997, *p* < 0.0001).

### 4.2. Progression of Cell Counts over Time

Several milk HRDCC values showed a particular dynamic over time. Granulocyte (Figure 1A) and lymphocyte (Figure 1B) counts fluctuated substantially at first, then leveled out after about 150 DIM. Macrophage counts remained roughly at the same level (Figure 1C). Percentages of gamma delta T cells declined (Figure 1D), whereas the percentages of CD4^+^ T cells followed the opposite trend (Figure 1E). For detailed graphs of all populations, please see the Supplementary Materials (Figures S2–S29). Percentages of CD8^+^ T cells were higher in the milk than in the blood (Figures S25 and S26). For B cells and NK cells, the opposite was observed (Figures S21, S22, S27 and S28). The mean viability of all milk cells, which was very low for the first sampling days, improved rapidly and further increased during the lactation (Figure S2).

**Figure 1:**
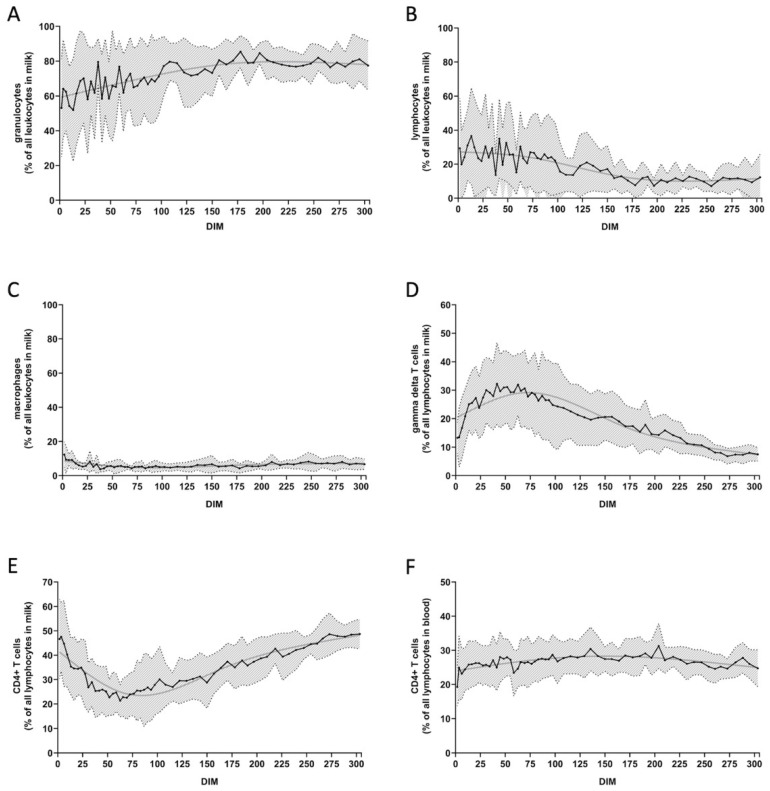
Mean percentages of cell subpopulations during the course of lactation, with standard deviation and smoothing spline. On average, there are 55 (52–57) data points each: (**A**) milk granulocytes; (**B**) milk lymphocytes; (**C**) milk macrophages; (**D**) milk gamma delta T cells; (**E**) milk CD4^+^ T cells; (**F**) blood CD4^+^ T cells.

Blood HRDCC values remained mostly constant over the course of lactation, albeit at an individual level for the respective cow. This can be seen, e.g., for blood CD4^+^ T cells (Figure 1F). The percentages for each cow fluctuated in a narrow range, but values of different cows varied widely, e.g., between cow 1 (mean = 34.7, SD = 2.9) and cow 6 (mean = 19.7, SD = 2.6). Detailed information on the count results of all cows is provided in Tables S5–S12.

### 4.3. Deviations in Cell Counts in Case of Disease

**Figure 2:**
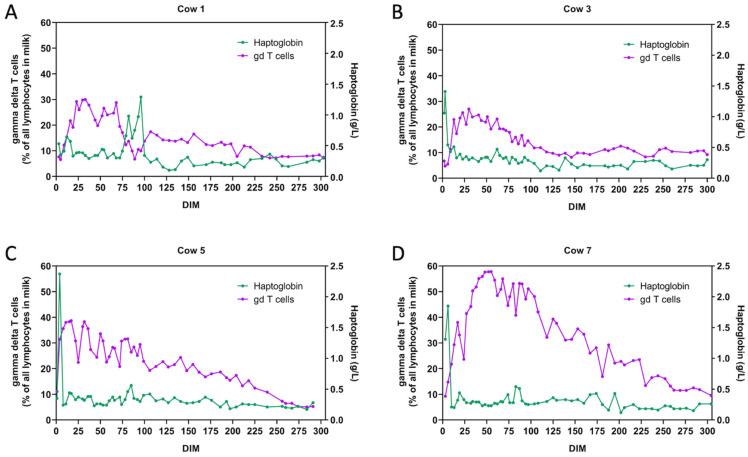
Relation of haptoglobin levels to percentages of gamma delta T cells during the course of lactation: (**A**) cow 1; (**B**) cow 3; (**C**) cow 5; (**D**) cow 7.

Some cows sickened occasionally during the 305-day study. Since haptoglobin levels were measured, we could not only detect clinical diseases but also subclinical inflammations. For example, cow 1 had a claw lesion that resulted in an increase in haptoglobin between 75 and 96 DIM, cow 3 developed mastitis directly after calving, and cow 7 suffered from a puerperal infection. All sickened cows were treated properly by a veterinarian. Cow 5 had elevated haptoglobin levels on the first sampling days without showing any clinical signs, probably due to smaller calving injuries. During these phases of inflammation, the percentages of milk gamma delta T cells seemed to decrease (Figure 2), accompanied by an increase in milk CD4^+^ T cells (Figure S23). No correlation between phases of inflammation and percentages of immature granulocytes (CD11b^low^) could be detected (Figures S12 and S13).

### 4.4. Unsupervised and Supervised Clustering

**Figure 3:**
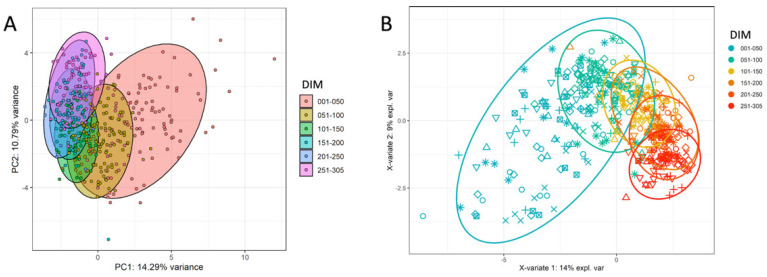
Results of different clustering methods, based on all measured parameters in the blood and milk. (**A**) PCA based on all parameters, shown here with the first principal component (PC1, 14.29% explained variance) and the second principal component (PC2, 10.79% explained variance). (**B**) sPLS-DA based on all parameters, shown here with the first component (X-variate 1, 14% explained variance) and the second component (X-variate 2, 9% explained variance). (AUROC values: phase “001–050 DIM” vs. others: 0.985; phase “051–100 DIM” vs. others: 0.895; phase “101–150 DIM” vs. others: 0.893; phase “151–200 DIM” vs. others: 0.864; phase “201–250 DIM” vs. others: 0.9148; phase “251–305 DIM” vs. others: 0.9893).

When using all measured parameters from the milk HRDCCs, blood HRDCCs, and results from the external laboratory, a similar trend was visible in both the PCA and sPLS-DA for the separation of six different lactation phases (Figure 3). The first group (1–50 DIM) was more widely spread than the others. As the lactation progressed, the groups diverged from the first one. In the sPLS-DA, this resulted in a continuous trend over the whole lactation period and a clear separation between the first and the last lactation phase, as can be seen from the corresponding AUROC (area under the receiver operating characteristics) values. Among the most important parameters which caused the separation in the sPLS-DA were NEFA, bilirubin, the viability of milk cells, milk CD4^+^ T cells, and milk gamma delta T cells.

**Figure 4:**
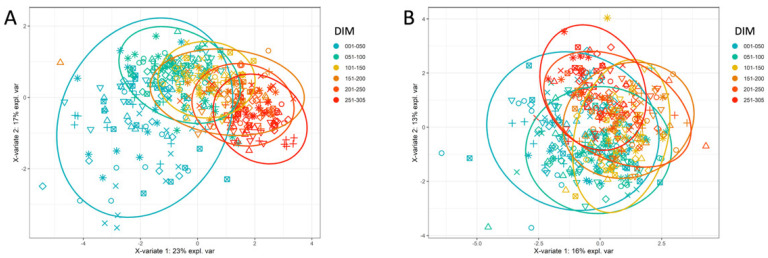
Results of sPLS-DA, based on different sets of parameters. (**A**) sPLS-DA based on milk HRDCC parameters, shown here with the first component (X-variate 1, 23% explained variance) and the second component (X-variate 2, 17% explained variance). (AUROC: phase “001–050 DIM” vs. others: 0.964; phase “051–100 DIM” vs. others: 0.83; phase “101–150 DIM” vs. others: 0.792; phase “151–200 DIM” vs. others: 0.732; phase “201–250 DIM” vs. others: 0.863; phase “251–305 DIM” vs. others: 0.965). (**B**) sPLS-DA based on blood HRDCC parameters, shown here with the first component (X-variate 1, 16% explained variance) and the second component (X-variate 2, 13% explained variance). (AUROC: phase “001–050 DIM” vs. others: 0.831; phase “051–100 DIM” vs. others: 0.784; phase “101–150 DIM” vs. others: 0.794; phase “151–200 DIM” vs. others: 0.778; phase “201–250 DIM” vs. others: 0.792; phase “251–305 DIM” vs. others: 0.906).

The best discriminating power was provided by the milk HRDCC parameters, which can be seen from the sPLS-DA results based on milk parameters only (Figure 4A). The first group was again more scattered than the others, and the clusters of the different groups followed each other closely. The most influential separating factors in the sPLS-DA were milk gamma delta T cells, the viability of milk cells, milk ncMac, milk NK cells, and milk CD4^+^ T cells. In contrast, no differences between the groups were seen when PCA and sPLS-DA were applied only on the blood HRDCC results (Figure 4B). The groups mostly overlapped for both clustering analyses. When the PCA and sPLS-DA were based solely on the results from the external laboratory, only the first group was partly separated from the rest (Figure S40). The remaining groups mainly overlapped.

## 5. Discussion

The SCC has been used for decades as an indicator of a potential intramammary inflammation and is still one of the most important parameters to control udder health [10]. Since several studies over the past twenty years have demonstrated the benefit of determining different milk immune cell populations [17,30,31], we recorded HRDCC values of several relevant (sub)populations over the whole lactation period. For this type of detailed immunomonitoring, flow cytometry is the method of choice as fluorochrome-conjugated antibodies allow accurate and efficient cell phenotyping. With the acquired comprehensive data, we could document cell count ranges of average healthy cows and identify potentially prognostic cell types that might prove to be useful as biomarkers in the case of disease.

There is no consensus in the literature about the relative distribution of the main immune cell populations, i.e., granulocytes, macrophages, and lymphocytes, in milk obtained from healthy udders, as reviewed by Halasa et al. [32]. In contrast to previous studies [18,31] that describe macrophages as the predominant population in milk, we detected this cell type only in consistently low percentages. In our study, granulocyte and lymphocyte percentages showed pronounced fluctuations in the first 150 DIM, but granulocytes were the predominant milk cell type in the mid to late phase of lactation, in agreement with Pilla et al. [16]. Other studies [17,18] considered an increase in granulocytes as a sign of inflammation. When assessing our data, it was noticeable that the percentages of milk gamma delta T cells and milk CD4^+^ T cells had a contrary progression throughout the lactation. This trend was also reflected in the sPLS-DA clustering, in which those T cell subsets were among the most important factors separating the different lactation phases. In agreement with our results, low percentages of milk CD4^+^ T cells after parturition as well as high numbers of CD4^+^ T cells at the end of lactation and after dry-off have been reported [33,34]. In ruminants, gamma delta T cells are much more frequent than in other species [35] and are likely to play an important role in immune defense. They function, for example, as a substantial regulatory T cell subpopulation as they can alter the CD4^+^ T cell response through TGF-β and IL-10 [36,37]. The characteristic distribution of those T cell subsets in early lactation might be caused by the metabolic condition of the cow. Most dairy cows undergo a phase of negative energy balance in the periparturient period, especially in the first few weeks after calving, when their feed intake cannot compensate the necessary energy consumption for increased milk production and body maintenance [38]. This phase of physiological imbalance can have a great impact on immune functions, as reviewed by Ingvartsen et al. [39]. However, we could not directly link the prominent changes in the milk DCC to elevated levels of metabolic parameters or high milk yields in the respective cows. In our study, percentages of CD8^+^ T cells were constantly higher in the milk than in the blood; the opposite was true for B cells. The same distributions were reported by Harp et al. [40]. No general assertations can be made about predominant lymphocyte subpopulations since we observed different incidences in our experimental cows’ data.

Since DCC results could be influenced by various factors, such as the sampling procedure, preparation protocol, or whether a preservative was used, it is difficult to compare cell counts determined by different methods [41,42,43]. Moreover, the cows in our study showed pronounced variations between individual subjects. Thus, it seems advisable to interpret cell count deviations based on cow-specific data, e.g., to compare DCC results of previous DHI tests of the same individual to detect cow-specific changes. Schwarz et al. [20] used the DHI results for the SCC and their newly implemented DCC to determine the udder health status of each cow and—based on that status—predicted its future state of health and whether this cow would be leaving the herd before the next DHI sampling day [44]. With this approach, the combined power of the SCC and DCC can compensate for the deficiencies of the individual parameters. If some type of DCC measurement could be implemented in automated milking systems in the future, even a day-by-day comparison of cow-specific results would be possible. Since our study is limited due to its small number of cows, larger studies including more cows of different breeds would be useful to obtain reference values.

In cases of inflammation, i.e., elevated haptoglobin levels, some of our cows showed an increase in milk CD4^+^ T cells, accompanied by a decrease in milk gamma delta T cell percentages. Similar findings have been described in previous studies. Rivas et al. [45] reported increased percentages of CD4^+^ T cells after inoculating healthy mammary gland quarters with *Staphylococcus aureus*. The authors suggested that this cell type played an important role in the early immunological response in the case of *S. aureus* mastitis. Consistent with these findings, Soltys et al. [46] stated elevated percentages of CD4^+^ T cells in the case of staphylococcal mastitis, but with a concurrent increase in gamma delta T cells and a decrease in CD8^+^ T cell percentages. In contrast, Souza et al. [18] found a prominent rise in the percentages of milk CD4^+^ lymphocytes only in the case of non-specific mastitis, which they defined as bacteriological culture-negative milk samples from mammary gland quarters with high qSCC values. The authors hypothesized that the present CD4^+^ T cells were able to lower the bacterial load in the milk of infected udder quarters, e.g., through leukocytosis caused by their production of IL-17A and INF-γ [47]. No correlation between the amount of immature granulocytes and haptoglobin levels could be found for the cows in our study, neither in the blood nor in the milk. This indicates that an increased percentage of immature granulocytes is not suitable as an early inflammation biomarker.

## 6. Conclusions

HRDCCs are a highly sensitive and reliable tool, albeit difficult to interpret due to high individual differences. In the present study, ratios of immune cell subpopulations and DCC patterns changed consistently over the entire lactation period. Milk gamma delta T cells and milk CD4^+^ T cells were notable due to their characteristic percentage fluctuations during the course of lactation as well as their shifts in the case of disease. To verify the use and validity of such prognostic cell types as inflammatory biomarkers in the daily diagnostic routine, extended studies are needed, which should include more cows of different breeds, of different performance levels, and in various health conditions. In general, it can be assumed that it would be beneficial to include some form of DCC in routine milk diagnostics, such as DHI testing or automatic milking systems, compared to the sole use of simple SCC measurements. Thus, the potential of milk as a liquid biopsy and valuable diagnostic and prognostic medium could be better exploited. The health and wellbeing of cows, milk quality, and cost efficiency of dairy farming could profit from it.

## Data Availability

The detailed results can be found in the Supplementary Materials.

## Supplementary Materials

The following supporting information can be downloaded at: https://www.mdpi.com/article/10.3390/ani12111339/s1, Table S1: Concentration of viability dye. Reagent was diluted in DPBS (Dulbecco’s Phosphate Buffered Saline, Sigma Aldrich, Co.), other reagents diluted in FACS buffer (DPBS with 2% fetal bovine serum (Sigma Aldrich, Co.) and 0.01% NaN3). Table S2: Concentrations of primary antibodies. Reagents were diluted in FACS buffer (DPBS with 2% fetal bovine serum (Sigma Aldrich, Co.) and 0.01% NaN3). Table S3: Concentrations of secondary antibodies. Reagents were diluted in FACS buffer (DPBS with 2% fetal bovine serum (Sigma Aldrich, Co.) and 0.01% NaN3). Table S4: Concentrations of isotype control antibodies. Table S5: Results of Cow 1. Percentages of live cells, percentages of different cell populations, levels of externally analyzed parameters, SSC values and milk yield. Table S6: Results of Cow 2. Percentages of live cells, percentages of different cell populations, levels of externally analyzed parameters, SSC values and milk yield. Table S7: Results of Cow 3. Percentages of live cells, percentages of different cell populations, levels of externally analyzed parameters, SSC values and milk yield. Table S8: Results of Cow 4. Percentages of live cells, percentages of different cell populations, levels of externally analyzed parameters, SSC values and milk yield. Table S9: Results of Cow 5. Percentages of live cells, percentages of different cell populations, levels of externally analyzed parameters, SSC values and milk yield. Table S10: Results of Cow 6. Percentages of live cells, percentages of different cell populations, levels of externally analyzed parameters, SSC values and milk yield. Table S11: Results of Cow 7. Percentages of live cells, percentages of different cell populations, levels of externally analyzed parameters, SSC values and milk yield. Table S12: Results of Cow 8. Percentages of live cells, percentages of different cell populations, levels of externally analyzed parameters, SSC values and milk yield. Figure S1: Exemplary gating strategy. (A) FSC-A vs. SSC-A, milk cells (A1), blood cells (A2); (B) Doublet discrimination, milk cells (B1) and blood cells (B2); (C) Determination of viability in single cells, milk (C1) and blood (C2);(D) Pan leukocyte marker CD45 vs. SSC-A in live cells, milk (D1) and blood (D2); (E1) Pan cytokeratin marker vs. SSC-A in CD45- cells, milk; (F) Autofluorescence (filter 525/50 nm) vs. SSC-A in granulocytes, milk (F1) and blood (F2); (G) CD11b vs. SSC-A in granulocytes, milk (G1) and blood (G2); (H) CD14 vs. CD16 in monocytes/macrophages, milk (H1) and blood (H2); (I) gdTCR vs. CD335 in lymphocytes, milk (I1) and blood (I2); (J) CD4 vs. CD8 in rest1, milk (J1) and blood (J2); (K) CD21 vs. SSC-A in rest2, milk (K1) and blood (K2). Figure S2: Progression of live cells in milk during the course of lactation, shown as percentage of total events. A: Mean percentages with SD and smoothing spline. B–I: Results of Cows 1–8; standard curves were interpolated (third order polynomial, 95% CI), outliers were excluded (Q = 1%) and marked (☐). Figure S3: Progression of live cells in blood during the course of lactation, shown as percentage of total events. A: Mean percentages with SD and smoothing spline. B–I: Results of Cows 1–8; standard curves were interpolated (third order polynomial, 95% CI), outliers were excluded (Q = 1%) and marked (☐). Figure S4: Progression of granulocytes in milk during the course of lactation, shown as percentage of all viable leukocytes. A: Mean percentages with SD and smoothing spline. B–I: Results of Cows 1–8; standard curves were interpolated (third order polynomial, 95% CI), outliers were excluded (Q = 1%) and marked (☐). Figure S5: Progression of granulocytes in blood during the course of lactation, shown as percentage of all viable leukocytes. A: Mean percentages with SD and smoothing spline. B–I: Results of Cows 1–8; standard curves were interpolated (third order polynomial, 95% CI), outliers were excluded (Q = 1%) and marked (☐). Figure S6: Progression of macrophages in milk during the course of lactation, shown as percentage of all viable leukocytes. A: Mean percentages with SD and smoothing spline. B–I: Results of Cows 1–8; standard curves were interpolated (third order polynomial, 95% CI), outliers were excluded (Q = 1%) and marked (☐). Figure S7: Progression of monocytes in blood during the course of lactation, shown as percentage of all viable leukocytes. A: Mean percentages with SD and smoothing spline. B–I: Results of Cows 1–8; standard curves were interpolated (third order polynomial, 95% CI), outliers were excluded (Q = 1%) and marked (☐). Figure S8: Progression of lymphocytes in milk during the course of lactation, shown as percentage of all viable leukocytes. A: Mean percentages with SD and smoothing spline. B–I: Results of Cows 1–8; standard curves were interpolated (third order polynomial, 95% CI), outliers were excluded (Q = 1%) and marked (☐). Figure S9: Progression of lymphocytes in blood during the course of lactation, shown as percentage of all viable leukocytes. A: Mean percentages with SD and smoothing spline. B–I: Results of Cows 1–8; standard curves were interpolated (third order polynomial, 95% CI), outliers were excluded (Q = 1%) and marked (☐). Figure S10: Progression of eosinophils in milk during the course of lactation, shown as percentage of all viable leukocytes. A: Mean percentages with SD and smoothing spline. B–I: Results of Cows 1–8; standard curves were interpolated (third order polynomial, 95% CI), outliers were excluded (Q = 1%) and marked (☐). Figure S11: Progression of eosinophils in blood during the course of lactation, shown as percentage of all viable leukocytes. A: Mean percentages with SD and smoothing spline. B–I: Results of Cows 1–8; standard curves were interpolated (third order polynomial, 95% CI), outliers were excluded (Q = 1%) and marked (☐). Figure S12: Progression of immature granulocytes in milk during the course of lactation, shown as percentage of all viable granulocytes. A: Mean percentages with SD and smoothing spline. B–I: Results of Cows 1–8; standard curves were interpolated (third order polynomial, 95% CI), outliers were excluded (Q = 1%) and marked (☐). Figure S13: Progression of immature granulocytes in blood during the course of lactation, shown as percentage of all viable granulocytes. A: Mean percentages with SD and smoothing spline. B–I: Results of Cows 1–8; standard curves were interpolated (third order polynomial, 95% CI), outliers were excluded (Q = 1%) and marked (☐). Figure S14: Progression of classical macrophages in milk during the course of lactation, shown as percentage of all viable macrophages. A: Mean percentages with SD and smoothing spline. B–I: Results of Cows 1–8; standard curves were interpolated (third order polynomial, 95% CI), outliers were excluded (Q = 1%) and marked (☐). Figure S15: Progression of nonclassical macrophages in milk during the course of lactation, shown as percentage of all viable macrophages. A: Mean percentages with SD and smoothing spline. B–I: Results of Cows 1–8; standard curves were interpolated (third order polynomial, 95% CI), outliers were excluded (Q = 1%) and marked (☐). Figure S16: Progression of classical monocytes in blood during the course of lactation, shown as percentage of all viable monocytes. A: Mean percentages with SD and smoothing spline. B–I: Results of Cows 1–8; standard curves were interpolated (third order polynomial, 95% CI), outliers were excluded (Q = 1%) and marked (☐). Figure S17: Progression of intermediate monocytes in blood during the course of lactation, shown as percentage of all viable monocytes. A: Mean percentages with SD and smoothing spline. B–I: Results of Cows 1–8; standard curves were interpolated (third order polynomial, 95% CI), outliers were excluded (Q = 1%) and marked (☐). Figure S18: Progression of nonclassical monocytes in blood during the course of lactation, shown as percentage of all viable monocytes. A: Mean percentages with SD and smoothing spline. B–I: Results of Cows 1–8; standard curves were interpolated (third order polynomial, 95% CI), outliers were excluded (Q = 1%) and marked (☐). Figure S19: Progression of gamma delta T cells in milk during the course of lactation, shown as percentage of all viable lymphocytes. A: Mean percentages with SD and smoothing spline. B–I: Results of Cows 1–8; standard curves were interpolated (third order polynomial, 95% CI), outliers were excluded (Q = 1%) and marked (☐). Figure S20: Progression of gamma delta T cells in blood during the course of lactation, shown as percentage of all viable lymphocytes. A: Mean percentages with SD and smoothing spline. B–I: Results of Cows 1–8; standard curves were interpolated (third order polynomial, 95% CI), outliers were excluded (Q = 1%) and marked (☐). Figure S21: Progression of NK cells in milk during the course of lactation, shown as percentage of all viable lymphocytes. A: Mean percentages with SD and smoothing spline. B–I: Results of Cows 1–8; standard curves were interpolated (third order polynomial, 95% CI), outliers were excluded (Q = 1%) and marked (☐). Figure S22: Progression of NK cells in milk during the course of lactation, shown as percentage of all viable lymphocytes. A: Mean percentages with SD and smoothing spline. B–I: Results of Cows 1–8; standard curves were interpolated (third order polynomial, 95% CI), outliers were excluded (Q = 1%) and marked (☐). Figure S23: Progression of CD4^+^ T cells in milk during the course of lactation, shown as percentage of all viable lymphocytes. A: Mean percentages with SD and smoothing spline. B–I: Results of Cows 1–8; standard curves were interpolated (third order polynomial, 95% CI), outliers were excluded (Q = 1%) and marked (☐). Figure S24: Progression of CD4^+^ T cells in blood during the course of lactation, shown as percentage of all viable lymphocytes. A: Mean percentages with SD and smoothing spline. B–I: Results of Cows 1–8; standard curves were interpolated (third order polynomial, 95% CI), outliers were excluded (Q = 1%) and marked (☐). Figure S25: Progression of CD8^+^ T cells in milk during the course of lactation, shown as percentage of all viable lymphocytes. A: Mean percentages with SD and smoothing spline. B–I: Results of Cows 1–8; standard curves were interpolated (third order polynomial, 95% CI), outliers were excluded (Q = 1%) and marked (☐). Figure S26: Progression of CD8^+^ T cells in blood during the course of lactation, shown as percentage of all viable lymphocytes. A: Mean percentages with SD and smoothing spline. B–I: Results of Cows 1–8; standard curves were interpolated (third order polynomial, 95% CI), outliers were excluded (Q = 1%) and marked (☐). Figure S27: Progression of B cells in milk during the course of lactation, shown as percentage of all viable lymphocytes. A: Mean percentages with SD and smoothing spline. B–I: Results of Cows 1–8; standard curves were interpolated (third order polynomial, 95% CI), outliers were excluded (Q = 1%) and marked (☐). Figure S28: Progression of B cells in blood during the course of lactation, shown as percentage of all viable lymphocytes. A: Mean percentages with SD and smoothing spline. B–I: Results of Cows 1–8; standard curves were interpolated (third order polynomial, 95% CI), outliers were excluded (Q = 1%) and marked (☐). Figure S29: Progression of MEC in milk during the course of lactation, shown as percentage of total events. A: Mean percentages with SD and smoothing spline. B–I: Results of Cows 1–8; standard curves were interpolated (third order polynomial, 95% CI), outliers were excluded (Q = 1%) and marked (☐). Figure S30: Haptoglobin levels in blood during the course of lactation. A: Mean percentages with SD and smoothing spline. B–I: Results of Cows 1–8; standard curves were interpolated (third order polynomial, 95% CI), outliers were excluded (Q = 1%) and marked (☐). Figure S31: β-HBA levels in blood during the course of lactation. A: Mean percentages with SD and smoothing spline. B–I: Results of Cows 1–8; standard curves were interpolated (third order polynomial, 95% CI), outliers were excluded (Q = 1%) and marked (☐). Figure S32: NEFA levels in blood during the course of lactation. A: Mean percentages with SD and smoothing spline. B–I: Results of Cows 1–8; standard curves were interpolated (third order polynomial, 95% CI), outliers were excluded (Q = 1%) and marked (☐). Figure S33: Bilirubin levels in blood during the course of lactation. A: Mean percentages with SD and smoothing spline. B–I: Results of Cows 1–8; standard curves were interpolated (third order polynomial, 95% CI), outliers were excluded (Q = 1%) and marked (☐). Figure S34: GLDH levels in blood during the course of lactation. A: Mean percentages with SD and smoothing spline. B–I: Results of Cows 1–8; standard curves were interpolated (third order polynomial, 95% CI), outliers were excluded (Q = 1%) and marked (☐). Figure S35: Calcium levels in blood during the course of lactation. A: Mean percentages with SD and smoothing spline. B–I: Results of Cows 1–8; standard curves were interpolated (third order polynomial, 95% CI), outliers were excluded (Q = 1%) and marked (☐). Figure S36: SCC values in milk during the course of lactation. A: Mean percentages with SD and smoothing spline. B–I: Results of Cows 1–8; standard curves were interpolated (third order polynomial, 95% CI), outliers were excluded (Q = 1%) and marked (☐). Figure S37: Milk yields at morning milking, during the course of lactation. A: Mean percentages with SD and smoothing spline. B–I: Results of Cows 1–8; standard curves were interpolated (third order polynomial, 95% CI), outliers were excluded (Q = 1%) and marked (☐). Figure S38: Results of PCA, based on milk HRDCC parameters. Figure S39: Results of PCA, based on blood HRDCC parameters. Figure S40: Results of clustering methods, based on parameters analyzed by external laboratory. A: PCA based on parameters analyzed by external laboratory. B: sPLS-DA based on parameters analyzed by external laboratory (AUROC: phase “001–050 DIM” vs. others: 0.977, phase “051–100 DIM” vs. others: 0.77, phase “101–150 DIM” vs. others: 0.851, phase “151–200 DIM” vs. others: 0.832, phase “201–250 DIM” vs. others: 0.878, phase “251–305 DIM” vs. others: 0.858).
