# Supplementary material for: Using High-Resolution Differential Cell Counts (HRDCCs) in Bovine Milk and Blood to Monitor the Immune Status over the Entire Lactation Period
Source: Animals (Basel). 2022 May 24;12(11):1339. doi: 10.3390/ani12111339 (PMC9179238; doi:10.3390/ani12111339)
Supplement: Supplementary file 1 [file animals-12-01339-s001.zip › animals-1720451-supplementary-TableS1-S12.pdf]

Supplementary Tables

Using high-resolution differential cell counts in bovine milk and blood to monitor the immune status over the entire lactation period

Sabine Farschtschi*, Alex Hildebrandt, Martin Mattes, Benedikt Kirchner, Michael W. Pfaffl.

Division of Animal Physiology and Immunology, TUM School of Life Sciences, Technical University of Munich, Freising, Germany.

*Corresponding author: sabine.farschtschi@tum.de

Table S1. **Concentration of viability dye.** Reagent was diluted in DPBS (Dulbecco's Phosphate Buffered Saline, Sigma Aldrich, Co.), other reagents diluted in FACS buffer (DPBS with 2% fetal bovine serum (Sigma Aldrich, Co.) and 0.01 % NaN_3).

Zombie NIR Fixable Viability Kit (Biolegend, Inc., San Diego, USA)	1:800

Table S2. **Concentrations of primary antibodies.** Reagents were diluted in FACS buffer (DPBS with 2% fetal bovine serum (Sigma Aldrich, Co.) and 0.01 % NaN_3).

Mouse anti-sheep CD45, Biotin, IgG1 (concentration 0.1 mg/ml), clone 1.11.32 (BioRad Laboratories Inc., Hercules, USA)	1:250
Mouse anti-bovine CD11b, unlabeled, IgG2b (concentration is 1.0 mg/ml), clone MM10A (Monoclonal Antibody Center, Washington State University, USA)	1:400
Mouse anti-human CD14, BV711, IgG2a (concentration 0.2 mg/ml), clone M5E2 (Becton, Dickinson and Company, Franklin Lakes, USA)	1:66

Mouse anti-human CD16, FITC, IgG2a (concentration 0.1mg/ml), clone KD1 (BioRad Laboratories Inc., Hercules, USA)	1:166
Cytokeratin pan monoclonal antibody, APC, IgG1 (concentration 0.1 mg/ml), clone C-11 (Thermo Fisher Scientific Inc., Waltham, USA)	1:400
Mouse anti-bovine CD4, Alexa Fluor 647, IgG2a (concentration 0.05 mg/ml), clone CC8 (BioRad Laboratories Inc., Hercules, USA)	1:250
Mouse anti-bovine CD8, unlabeled, IgG3 (concentration 1.0 mg/ml), clone CACT130A (Monoclonal Antibody Center, Washington State University, USA)	1:250
Mouse anti-bovine CD21, PE-Cy7, IgG1 (concentration 0.1 mg/ml), clone LT21 (EXBIO Praha, a.s., Czech Republic)	1:200
Mouse anti-bovine CD335, PE, IgG1 (concentration 1.0 mg/ml), clone AKS1 (BioRad Laboratories Inc. Hercules, USA)	1:20
Mouse anti-bovine γδ TCR1-N24 δ chain, IgG2b (concentration 1.0 mg/ml), clone GB21A (Monoclonal Antibody Center, Washington State University, USA)	1:250

Table S3. **Concentrations of secondary antibodies.** Reagents were diluted in FACS buffer (DPBS with 2% fetal bovine serum (Sigma Aldrich, Co.) and 0.01 % NaN_3).

Streptavidin (concentration 0.1 mg/ml) BV786 (Becton, Dickinson and Company, Franklin Lakes, USA)	1:800
Rat anti-mouse IgG2b (concentration 0.2 mg/ml), BV605, clone R12-3 (Becton, Dickinson and Company, Franklin Lakes, USA)	1:1,000
Rat anti-mouse IgG3 (concentration 0.2 mg/ml), BV421, clone R40-82 (Becton, Dickinson and Company, Franklin Lakes, USA)	1:400
Rat anti-mouse IgG2b (concentration 0.5 mg/ml), FITC, clone m2b-25G4 (Thermo Fisher Scientific Inc., Waltham, USA)	1:1,000

Table S4. **Concentrations of isotype control antibodies.**

Mouse IgG2a (concentration 0.05 mg/ml), Alexa Fluor 647 (BioRad Laboratories Inc., Hercules, USA)	1:250
Mouse IgG2a (concentration 0.2 mg/ml), BV711 (Becton, Dickinson and Company, Franklin Lakes, USA)	1:66
Mouse IgG2a (concentration 0.1 mg/ml), FITC (BioRad Laboratories Inc., Hercules, USA)	1:166
Mouse IgG1 (concentration 0.1 mg/ml), PE-Cy7 (EXBIO Praha, a.s., Czech Republic)	1:200
Mouse IgG1 (concentration 1.0 mg/ml), PE (BioRad Laboratories Inc., Hercules, USA)	1:20
Mouse IgG1 (0.2 mg/ml), APC (Thermo Fisher Scientific Inc., Waltham, USA)	1:800

Table S5. **Results of Cow 1.** Percentages of live cells, percentages of different cell populations, levels of externally analyzed parameters, SSC and milk yield.

Parameter	n	Mean	SD	Minimum	Maximum
Live cells in milk (% of total events)	52	44.9	16.4	9.7	68.7
Granulocytes in milk (% of all viable leukocytes)	52	81.1	5.9	67.1	96.2
Macrophages in milk (% of all viable leukocytes)	52	6.3	2.2	2.4	10.4
Lymphocytes in milk (% of all viable leukocytes)	52	9.3	5.6	0.6	25.3
Immature granulocytes in milk (% of all viable granulocytes)	52	0.9	0.4	0.4	2.3
Eosinophils in milk (% of all viable leukocytes)	52	1.0	0.5	0.1	2.6
Nonclassical macrophages in milk (% of all viable macrophages)	51	0.5	0.4	0.1	1.6

Classical macrophages in milk (% of all viable macrophages)	51	62.5	15.0	29.6	85.0
Mammary epithelial cells in milk (% of total events)	37	0.8	0.5	0.2	2.0
NK cells in milk (% of all viable lymphocytes)	48	0.5	0.4	0.2	2.1
Gamma delta T cells in milk (% of all viable lymphocytes)	52	15.2	7.1	6.5	30.0
$CD4^+$ T cells in milk (% of all viable lymphocytes)	51	39.2	9.5	19.5	54.9
$CD8^+$ T cells in milk (% of all viable lymphocytes)	52	33.6	5.5	20.1	44.6
B cells in milk (% of all viable lymphocytes)	51	1.0	0.9	0.2	4.3
Live cells in blood (% of total events)	51	58.5	11.4	36.5	82.0
Granulocytes in blood (% of all viable leukocytes)	51	55.8	8.6	35.9	74.5
Monocytes in blood (% of all viable leukocytes)	51	8.3	2.4	5.5	20.2
Lymphocytes in blood (% of all viable leukocytes)	51	34.3	8.4	15.3	56.1
Immature granulocytes in blood (% of all viable granulocytes)	51	0.7	0.5	0.1	3.1
Eosinophils in blood (% of all viable leukocytes)	51	4.7	1.9	0.9	10.5
Nonclassical monocytes in blood (% of all viable monocytes)	50	3.2	1.4	0.6	7.4
Intermediate monocytes in blood (% of all viable monocytes)	50	3.5	1.3	1.1	6.1
Classical monocytes in blood (% of all viable monocytes)	50	83.1	3.8	73.2	89.4
NK cells in blood (% of all viable lymphocytes)	50	3.2	1.0	1.3	5.4
Gamma delta T cells in blood (% of all viable lymphocytes)	52	5.2	1.7	3.3	11.2
$CD4^+$ T cells in blood (% of all viable lymphocytes)	51	34.6	2.9	28.8	40.4

$CD8^+$ T cells in blood (% of all viable lymphocytes)	52	5.1	1.2	3.3	10.5
B cells in blood (% of all viable lymphocytes)	51	28.4	2.4	23.2	34.2
Haptoglobin (g/l)	52	0.4	0.2	0.1	1.3
Calcium (mmol/l)	52	2.5	0.1	2.3	2.7
GLDH (U/l)	52	21.1	25.8	2.7	128.1
β-HBA (mmol/l)	52	0.9	0.5	0.4	2.8
NEFA (mmol/l)	52	0.7	0.6	0.1	2.5
Bilirubin (µmol/l)	52	3.6	3.4	0.5	16.6
SCC (cells/ml)	52	72258	70642	13300	339000
Milk yield at morning milking (l)	46	16.6	2.8	10.1	21.3

Table S6. **Results of Cow 2.** Percentages of live cells, percentages of different cell populations, levels of externally analyzed parameters, SSC and milk yield.

Parameter	n	Mean	SD	Minimum	Maximum
Live cells in milk (% of total events)	55	54.3	15.6	12.4	78.5
Granulocytes in milk (% of all viable leukocytes)	55	86.3	9.9	46.1	95.3
Macrophages in milk (% of all viable leukocytes)	55	3.6	1.6	1.0	8.3
Lymphocytes in milk (% of all viable leukocytes)	55	7.3	8.3	0.7	49.9
Immature granulocytes in milk (% of all viable granulocytes)	55	0.7	0.4	0.1	1.6

Eosinophils in milk (% of all viable leukocytes)	55	1.1	0.9	0.0	5.3
Nonclassical macrophages in milk (% of all viable macrophages)	46	0.7	0.7	0.0	3.3
Classical macrophages in milk (% of all viable macrophages)	46	45.8	13.6	13.9	74.0
Mammary epithelial cells in milk (% of total events)	39	0.7	0.3	0.2	1.6
NK cells in milk (% of all viable lymphocytes)	53	0.7	0.7	0.1	4.5
Gamma delta T cells in milk (% of all viable lymphocytes)	55	11.5	4.6	4.2	21.6
$CD4^+$ T cells in milk (% of all viable lymphocytes)	54	40.7	10.7	16.7	62.0
$CD8^+$ T cells in milk (% of all viable lymphocytes)	55	34.2	6.4	20.3	53.1
B cells in milk (% of all viable lymphocytes)	54	0.5	0.4	0.1	1.6
Live cells in blood (% of total events)	55	62.8	9.8	36.0	78.3
Granulocytes in blood (% of all viable leukocytes)	55	48.7	7.4	34.0	70.9
Monocytes in blood (% of all viable leukocytes)	55	7.2	1.5	4.2	10.1
Lymphocytes in blood (% of all viable leukocytes)	55	42.9	7.4	22.8	56.4
Immature granulocytes in blood (% of all viable granulocytes)	54	0.9	0.6	0.2	2.8
Eosinophils in blood (% of all viable leukocytes)	54	1.8	0.7	0.5	4.4
Nonclassical monocytes in blood (% of all viable monocytes)	46	3.6	1.5	0.6	7.8
Intermediate monocytes in blood (% of all viable monocytes)	46	4.4	1.5	2.0	8.5
Classical monocytes in blood (% of all viable monocytes)	46	79.5	4.9	67.7	89.7
NK cells in blood (% of all viable lymphocytes)	54	1.7	0.4	0.9	3.4

Gamma delta T cells in blood (% of all viable lymphocytes)	55	5.7	0.6	4.2	6.7
$CD4^+$ T cells in blood (% of all viable lymphocytes)	54	26.8	3.5	14.4	42.5
$CD8^+$ T cells in blood (% of all viable lymphocytes)	55	8.5	1.2	5.2	12.8
B cells in blood (% of all viable lymphocytes)	54	37.7	3.8	23.2	54.7
Haptoglobin (g/l)	55	0.4	0.3	0.2	1.5
Calcium (mmol/l)	55	2.5	0.1	2.2	2.9
GLDH (U/l)	55	28.7	12.4	8.3	72.0
β-HBA (mmol/l)	55	0.6	0.2	0.3	1.0
NEFA (mmol/l)	55	0.3	0.2	0.1	1.3
Bilirubin (µmol/l)	55	2.8	1.2	1.0	6.4
SCC (cells/ml)	55	38186	38735	4900	264000
Milk yield at morning milking (l)	54	21.2	4.7	10.8	26.1

Table S7. **Results of Cow 3.** Percentages of live cells, percentages of different cell populations, levels of externally analyzed parameters, SSC and milk yield.

Parameter	n	Mean	SD	Minimum	Maximum
Live cells in milk (% of total events)	53	46.8	16.7	16.4	76.5
Granulocytes in milk (% of all viable leukocytes)	53	65.1	24.9	7.6	94.7
Macrophages in milk (% of all viable leukocytes)	53	6.8	2.7	1.5	15.4

Lymphocytes in milk (% of all viable leukocytes)	53	25.1	25.6	1.5	87.4
Immature granulocytes in milk (% of all viable granulocytes)	53	3.0	3.4	0.5	18.4
Eosinophils in milk (% of all viable leukocytes)	53	0.9	0.4	0.1	1.9
Nonclassical macrophages in milk (% of all viable macrophages)	52	0.7	2.9	0.0	21.0
Classical macrophages in milk (% of all viable macrophages)	52	56.9	17.0	15.4	82.4
Mammary epithelial cells in milk (% of total events)	38	1.2	0.8	0.1	3.2
NK cells in milk (% of all viable lymphocytes)	49	0.5	0.2	0.2	0.9
Gamma delta T cells in milk (% of all viable lymphocytes)	53	14.2	6.0	4.7	27.0
$CD4^+$ T cells in milk (% of all viable lymphocytes)	52	40.8	9.6	27.3	65.0
$CD8^+$ T cells in milk (% of all viable lymphocytes)	53	31.4	7.4	7.4	45.0
B cells in milk (% of all viable lymphocytes)	52	0.3	0.3	0.0	1.9
Live cells in blood (% of total events)	53	64.3	8.6	41.7	81.3
Granulocytes in blood (% of all viable leukocytes)	53	47.8	7.2	32.2	70.6
Monocytes in blood (% of all viable leukocytes)	53	7.8	3.4	5.0	26.8
Lymphocytes in blood (% of all viable leukocytes)	53	42.8	8.2	16.2	57.6
Immature granulocytes in blood (% of all viable granulocytes)	53	0.9	1.1	0.1	8.0
Eosinophils in blood (% of all viable leukocytes)	53	2.2	1.9	0.2	9.7
Nonclassical monocytes in blood (% of all viable monocytes)	52	2.4	1.2	0.0	6.4
Intermediate monocytes in blood (% of all viable monocytes)	52	3.2	1.4	0.6	9.3

Classical monocytes in blood (% of all viable monocytes)	52	81.5	3.4	73.3	86.3
NK cells in blood (% of all viable lymphocytes)	50	2.4	0.6	0.2	3.4
Gamma delta T cells in blood (% of all viable lymphocytes)	52	12.0	2.5	6.5	16.7
$CD4^+$ T cells in blood (% of all viable lymphocytes)	51	28.5	4.2	20.7	38.9
$CD8^+$ T cells in blood (% of all viable lymphocytes)	52	9.9	2.0	6.6	16.2
B cells in blood (% of all viable lymphocytes)	51	23.6	3.1	14.8	35.4
Haptoglobin (g/l)	52	0.3	0.2	0.1	1.4
Calcium (mmol/l)	52	2.6	0.1	2.1	2.9
GLDH (U/l)	52	18.6	11.7	7.1	77.5
β-HBA (mmol/l)	52	0.7	0.2	0.4	1.4
NEFA (mmol/l)	52	0.3	0.2	0.1	0.9
Bilirubin (µmol/l)	52	1.9	1.4	0.2	6.9
SCC (cells/ml)	53	83649	177042	6800	1050000
Milk yield at morning milking (l)	48	20.7	3.7	11.9	26.5

Table S8. **Results of Cow 4.** Percentages of live cells, percentages of different cell populations, levels of externally analyzed parameters, SSC and milk yield.

Parameter	n	Mean	SD	Minimum	Maximum
Live cells in milk (% of total events)	54	51.7	17.1	3.8	84.5

Granulocytes in milk (% of all viable leukocytes)	53	65.6	14.8	26.2	90.0
Macrophages in milk (% of all viable leukocytes)	54	10.5	4.6	3.9	22.7
Lymphocytes in milk (% of all viable leukocytes)	54	20.9	13.3	2.0	62.1
Immature granulocytes in milk (% of all viable granulocytes)	54	2.0	1.1	0.2	5.0
Eosinophils in milk (% of all viable leukocytes)	53	1.0	0.5	0.2	2.4
Nonclassical macrophages in milk (% of all viable macrophages)	49	0.5	0.5	0.0	2.7
Classical macrophages in milk (% of all viable macrophages)	49	73.3	12.4	33.8	98.1
Mammary epithelial cells in milk (% of total events)	36	1.2	0.6	0.2	3.5
NK cells in milk (% of all viable lymphocytes)	53	0.6	0.3	0.2	1.7
Gamma delta T cells in milk (% of all viable lymphocytes)	55	24.3	11.5	7.4	45.5
$CD4^+$ T cells in milk (% of all viable lymphocytes)	54	31.5	9.5	16.6	51.0
$CD8^+$ T cells in milk (% of all viable lymphocytes)	55	33.1	5.5	18.5	49.3
B cells in milk (% of all viable lymphocytes)	53	0.3	0.3	0.0	1.1
Live cells in blood (% of total events)	55	44.4	8.5	29.7	61.3
Granulocytes in blood (% of all viable leukocytes)	55	49.1	6.9	33.0	62.3
Monocytes in blood (% of all viable leukocytes)	55	10.5	2.9	4.6	22.7
Lymphocytes in blood (% of all viable leukocytes)	55	39.3	7.1	21.1	56.3
Immature granulocytes in blood (% of all viable granulocytes)	55	0.5	0.3	0.1	1.8
Eosinophils in blood (% of all viable leukocytes)	55	11.4	6.4	1.9	33.6

Nonclassical monocytes in blood (% of all viable monocytes)	49	2.8	1.2	0.1	5.1
Intermediate monocytes in blood (% of all viable monocytes)	49	3.4	1.7	1.2	12.3
Classical monocytes in blood (% of all viable monocytes)	49	85.3	3.1	76.7	91.5
NK cells in blood (% of all viable lymphocytes)	54	3.2	0.7	0.8	4.6
Gamma delta T cells in blood (% of all viable lymphocytes)	55	11.6	1.5	6.7	14.6
$CD4^+$ T cells in blood (% of all viable lymphocytes)	54	30.7	3.7	20.6	37.4
$CD8^+$ T cells in blood (% of all viable lymphocytes)	55	7.9	1.5	5.6	11.2
B cells in blood (% of all viable lymphocytes)	53	23.0	3.2	18.9	37.9
Haptoglobin (g/l)	54	0.3	0.1	0.1	0.7
Calcium (mmol/l)	54	2.6	0.2	2.2	3.0
GLDH (U/l)	54	19.8	10.5	7.1	60.6
β-HBA (mmol/l)	54	0.7	0.3	0.4	1.6
NEFA (mmol/l)	54	0.4	0.2	0.2	1.2
Bilirubin (µmol/l)	54	2.4	1.7	0.8	8.7
SCC (cells/ml)	55	38933	31101	7100	200000
Milk yield at morning milking (l)	52	17.8	2.3	12.2	20.4

Table S9. **Results of Cow 5.** Percentages of live cells, percentages of different cell populations, levels of externally analyzed parameters, SSC and milk yield.

Parameter	n	Mean	SD	Minimum	Maximum
Live cells in milk (% of total events)	54	35.6	17.6	6.0	75.6
Granulocytes in milk (% of all viable leukocytes)	54	61.6	23.4	18.4	91.6
Macrophages in milk (% of all viable leukocytes)	54	5.1	3.2	2.0	22.5
Lymphocytes in milk (% of all viable leukocytes)	54	30.5	23.2	3.5	74.1
Immature granulocytes in milk (% of all viable granulocytes)	54	1.8	2.3	0.1	17.0
Eosinophils in milk (% of all viable leukocytes)	54	1.4	0.9	0.0	4.8
Nonclassical macrophages in milk (% of all viable macrophages)	46	1.1	1.1	0.0	4.8
Classical macrophages in milk (% of all viable macrophages)	46	63.7	12.5	20.9	82.7
Mammary epithelial cells in milk (% of total events)	38	0.8	0.5	0.1	3.4
NK cells in milk (% of all viable lymphocytes)	52	0.7	0.6	0.0	3.6
Gamma delta T cells in milk (% of all viable lymphocytes)	54	22.5	9.5	5.0	38.7
$CD4^+$ T cells in milk (% of all viable lymphocytes)	52	23.6	10.1	10.2	46.2
$CD8^+$ T cells in milk (% of all viable lymphocytes)	53	41.8	7.3	17.1	54.0
B cells in milk (% of all viable lymphocytes)	52	0.5	0.5	0.0	2.5
Live cells in blood (% of total events)	54	65.0	6.8	43.1	79.0
Granulocytes in blood (% of all viable leukocytes)	54	38.9	8.0	21.8	58.5
Monocytes in blood (% of all viable leukocytes)	54	6.0	2.2	2.3	18.3
Lymphocytes in blood (% of all viable leukocytes)	54	53.1	7.9	35.4	72.6

Immature granulocytes in blood (% of all viable granulocytes)	54	1.1	0.8	0.3	4.0
Eosinophils in blood (% of all viable leukocytes)	54	4.0	2.5	0.8	13.7
Nonclassical monocytes in blood (% of all viable monocytes)	46	3.8	1.8	0.7	10.0
Intermediate monocytes in blood (% of all viable monocytes)	46	4.4	1.9	0.4	10.9
Classical monocytes in blood (% of all viable monocytes)	46	79.8	5.3	64.9	89.0
NK cells in blood (% of all viable lymphocytes)	52	2.0	0.3	1.4	2.6
Gamma delta T cells in blood (% of all viable lymphocytes)	53	8.2	1.5	5.8	11.7
$CD4^+$ T cells in blood (% of all viable lymphocytes)	52	27.1	2.5	20.0	31.1
$CD8^+$ T cells in blood (% of all viable lymphocytes)	53	11.1	1.9	7.4	15.7
B cells in blood (% of all viable lymphocytes)	52	28.9	2.5	23.0	34.4
Haptoglobin (g/l)	54	0.3	0.3	0.2	2.4
Calcium (mmol/l)	53	2.6	0.2	1.7	2.9
GLDH (U/l)	54	21.6	7.2	11.1	48.2
β-HBA (mmol/l)	54	0.7	0.2	0.4	1.4
NEFA (mmol/l)	54	0.3	0.1	0.2	0.8
Bilirubin (µmol/l)	54	2.2	0.9	0.9	5.6
SCC (cells/ml)	54	24553	27132	1700	139000
Milk yield at morning milking (l)	51	18.0	3.0	9.0	21.4

Table S10. **Results of Cow 6.** Percentages of live cells, percentages of different cell populations, levels of externally analyzed parameters, SSC and milk yield.

Parameter	n	Mean	SD	Minimum	Maximum
Live cells in milk (% of total events)	57	51.8	15.1	9.6	80.0
Granulocytes in milk (% of all viable leukocytes)	57	71.8	13.2	31.9	96.0
Macrophages in milk (% of all viable leukocytes)	57	6.6	2.5	2.0	14.2
Lymphocytes in milk (% of all viable leukocytes)	57	18.4	11.3	0.7	49.2
Immature granulocytes in milk (% of all viable granulocytes)	57	1.3	1.6	0.1	11.3
Eosinophils in milk (% of all viable leukocytes)	57	1.2	1.1	0.0	6.3
Nonclassical macrophages in milk (% of all viable macrophages)	48	0.7	0.7	0.0	4.1
Classical macrophages in milk (% of all viable macrophages)	48	51.9	13.6	14.8	96.8
Mammary epithelial cells in milk (% of total events)	39	0.5	0.4	0.1	2.4
NK cells in milk (% of all viable lymphocytes)	54	0.4	0.2	0.2	1,3
Gamma delta T cells in milk (% of all viable lymphocytes)	57	24.2	13.3	3.7	47.6
$CD4^+$ T cells in milk (% of all viable lymphocytes)	57	29.0	12.8	13.4	53.2
$CD8^+$ T cells in milk (% of all viable lymphocytes)	57	36.1	4.2	23.2	42.8
B cells in milk (% of all viable lymphocytes)	55	0.4	0.2	0.0	1.1
Live cells in blood (% of total events)	57	57.4	8.6	35.6	78.7
Granulocytes in blood (% of all viable leukocytes)	57	38.2	7.3	23.9	62.6

Monocytes in blood (% of all viable leukocytes)	57	6.4	1.7	2.0	15.6
Lymphocytes in blood (% of all viable leukocytes)	57	54.0	7.0	33.7	65.5
Immature granulocytes in blood (% of all viable granulocytes)	57	1.3	1.6	0.1	9.8
Eosinophils in blood (% of all viable leukocytes)	57	4.0	3.6	0.9	19.2
Nonclassical monocytes in blood (% of all viable monocytes)	48	2.6	1.0	0.5	5.3
Intermediate monocytes in blood (% of all viable monocytes)	48	3.1	1.2	0.9	6.5
Classical monocytes in blood (% of all viable monocytes)	48	80.4	4.8	65.2	88.6
NK cells in blood (% of all viable lymphocytes)	56	2.2	0.4	1.2	3.8
Gamma delta T cells in blood (% of all viable lymphocytes)	57	7.3	0.8	5.6	9.4
$CD4^+$ T cells in blood (% of all viable lymphocytes)	57	19.8	2.6	14.2	26.9
$CD8^+$ T cells in blood (% of all viable lymphocytes)	57	9.8	1.7	6.7	13.6
B cells in blood (% of all viable lymphocytes)	55	37.3	3.1	28.1	44.1
Haptoglobin (g/l)	57	0.4	0.9	0.1	7.2
Calcium (mmol/l)	57	2.4	0.1	1.9	2.7
GLDH (U/l)	57	11.7	3.6	5.7	27.1
β-HBA (mmol/l)	57	0.5	0.1	0.1	0.9
NEFA (mmol/l)	57	0.3	0.2	0.1	1.0
Bilirubin (μmol/l)	57	2.0	1.3	0.1	7.0
SCC (cells/ml)	57	103144	90793	9700	482500

Milk yield at morning milking (l)	54	17.3	4.5	6.6	23.2

Table S11. **Results of Cow 7.** Percentages of live cells, percentages of different cell populations, levels of externally analyzed parameters, SSC and milk yield.

Parameter	n	Mean	SD	Minimum	Maximum
Live cells in milk (% of total events)	57	43.1	13.7	8.1	74.1
Granulocytes in milk (% of all viable leukocytes)	57	63.1	21.1	24.2	94.5
Macrophages in milk (% of all viable leukocytes)	57	5.5	2.3	2.1	13.6
Lymphocytes in milk (% of all viable leukocytes)	57	28.6	21.9	1.4	70.4
Immature granulocytes in milk (% of all viable granulocytes)	57	2.1	1.3	0.3	8.1
Eosinophils in milk (% of all viable leukocytes)	57	1.5	1.1	0.0	6.2
Nonclassical macrophages in milk (% of all viable macrophages)	49	0.7	0.9	0.0	3.2
Classical macrophages in milk (% of all viable macrophages)	49	68.9	12.6	16.9	86.0
Mammary epithelial cells in milk (% of total events)	37	1.4	0.8	0.2	3.3
NK cells in milk (% of all viable lymphocytes)	54	0.7	0.7	0.1	4.5
Gamma delta T cells in milk (% of all viable lymphocytes)	57	33.9	15.9	9.2	57.8
$CD4^+$ T cells in milk (% of all viable lymphocytes)	57	28.1	15.0	8.0	58.2
$CD8^+$ T cells in milk (% of all viable lymphocytes)	57	25.7	3.9	15.5	33.5
B cells in milk (% of all viable lymphocytes)	55	0.5	0.5	0.1	2.5

Live cells in blood (% of total events)	57	53.0	11.0	35.4	76.3
Granulocytes in blood (% of all viable leukocytes)	57	37.2	8.2	20.8	56.7
Monocytes in blood (% of all viable leukocytes)	57	5.8	2.0	1.8	13.5
Lymphocytes in blood (% of all viable leukocytes)	57	55.0	8.7	29.5	73.1
Immature granulocytes in blood (% of all viable granulocytes)	56	1.4	1.2	0.1	6.6
Eosinophils in blood (% of all viable leukocytes)	56	2.6	3.5	0.4	25.7
Nonclassical monocytes in blood (% of all viable monocytes)	49	3.6	1.5	0.8	6.4
Intermediate monocytes in blood (% of all viable monocytes)	49	3.1	1.4	1.3	9.0
Classical monocytes in blood (% of all viable monocytes)	49	77.6	6.5	57.7	90.4
NK cells in blood (% of all viable lymphocytes)	55	1.6	0.4	1.1	3.6
Gamma delta T cells in blood (% of all viable lymphocytes)	57	7.1	1.6	3.8	11.5
$CD4^+$ T cells in blood (% of all viable lymphocytes)	57	22.5	3.2	12.0	28.1
$CD8^+$ T cells in blood (% of all viable lymphocytes)	57	8.0	1.4	5.5	11.4
B cells in blood (% of all viable lymphocytes)	55	37.2	3.6	21.7	42.9
Haptoglobin (g/l)	57	0.3	0.3	0.1	1.9
Calcium (mmol/l)	57	2.7	0.1	2.2	3.0
GLDH (U/l)	57	32.0	14.3	5.5	73.2
β-HBA (mmol/l)	57	0.6	0.2	0.4	1.3
NEFA (mmol/l)	57	0.4	0.2	0.1	1.0

Bilirubin (µmol/l)	57	2.2	1.2	0.5	6.1
SCC (cells/ml)	57	50026	56210	4900	378666
Milk yield at morning milking (l)	54	17.9	1.6	14.1	21.3

Table S12. **Results of Cow 8.** Percentages of live cells, percentages of different cell populations, levels of externally analyzed parameters, SSC and milk yield.

Parameter	n	Mean	SD	Minimum	Maximum
Live cells in milk (% of total events)	56	55.8	16.8	9.4	81.6
Granulocytes in milk (% of all viable leukocytes)	56	82.0	15.1	28.3	97.0
Macrophages in milk (% of all viable leukocytes)	56	4.1	2.7	0.9	14.2
Lymphocytes in milk (% of all viable leukocytes)	56	10.7	12.2	1.0	60.2
Immature granulocytes in milk (% of all viable granulocytes)	56	1.1	1.8	0.1	13.1
Eosinophils in milk (% of all viable leukocytes)	56	1.0	0.9	0.0	4.0
Nonclassical macrophages in milk (% of all viable macrophages)	50	0.5	0.4	0.0	1.9
Classical macrophages in milk (% of all viable macrophages)	50	62.1	15.5	2.6	87.8
Mammary epithelial cells in milk (% of total events)	42	0.4	0.3	0.0	1.3
NK cells in milk (% of all viable lymphocytes)	53	1.2	0.7	0.3	3.2
Gamma delta T cells in milk (% of all viable lymphocytes)	56	21.6	8.0	4.7	34.1
$CD4^+$ T cells in milk (% of all viable lymphocytes)	56	35.3	8.3	21.8	58.9

CD8+ T cells in milk (% of all viable lymphocytes)	56	31.9	8.2	14.1	48.6
B cells in milk (% of all viable lymphocytes)	55	1.4	0.5	0.4	2.6
Live cells in blood (% of total events)	56	69.9	7.3	47.2	81.0
Granulocytes in blood (% of all viable leukocytes)	56	42.0	8.4	20.2	61.5
Monocytes in blood (% of all viable leukocytes)	56	8.1	2.1	3.3	17.2
Lymphocytes in blood (% of all viable leukocytes)	56	48.4	7.9	32.0	63.5
Immature granulocytes in blood (% of all viable granulocytes)	56	0.9	0.7	0.2	3.5
Eosinophils in blood (% of all viable leukocytes)	56	5.3	4.1	0.5	18.9
Nonclassical monocytes in blood (% of all viable monocytes)	49	3.1	1.4	1.0	6.8
Intermediate monocytes in blood (% of all viable monocytes)	49	3.9	1.4	1.6	6.8
Classical monocytes in blood (% of all viable monocytes)	49	80.1	4.2	67.0	87.7
NK cells in blood (% of all viable lymphocytes)	53	1.3	0.2	0.7	1.9
Gamma delta T cells in blood (% of all viable lymphocytes)	54	14.7	1.8	11.7	19.7
CD4+ T cells in blood (% of all viable lymphocytes)	54	25.2	3.9	12.9	32.5
CD8+ T cells in blood (% of all viable lymphocytes)	54	7.1	1.2	5.1	10.9
B cells in blood (% of all viable lymphocytes)	53	27.3	2.1	22.1	31.3
Haptoglobin (g/l)	56	0.3	0.2	0.1	1.3
Calcium (mmol/l)	56	2.6	0.1	2.2	2.9
GLDH (U/l)	56	17.2	14.4	3.8	66.6

β-HBA (mmol/l)	56	0.4	0.1	0.3	0.8
NEFA (mmol/l)	56	0.3	0.1	0.1	0.5
Bilirubin (µmol/l)	56	1.6	0.8	0.3	3.6
SCC (cells/ml)	56	96427	75640	35900	420000
Milk yield at morning milking (l)	53	11.6	1.3	8.1	14.3
