# Supplementary figures and images for: Using High-Resolution Differential Cell Counts (HRDCCs) in Bovine Milk and Blood to Monitor the Immune Status over the Entire Lactation Period
Source: Animals (Basel). 2022 May 24;12(11):1339. doi: 10.3390/ani12111339 (PMC9179238; doi:10.3390/ani12111339)

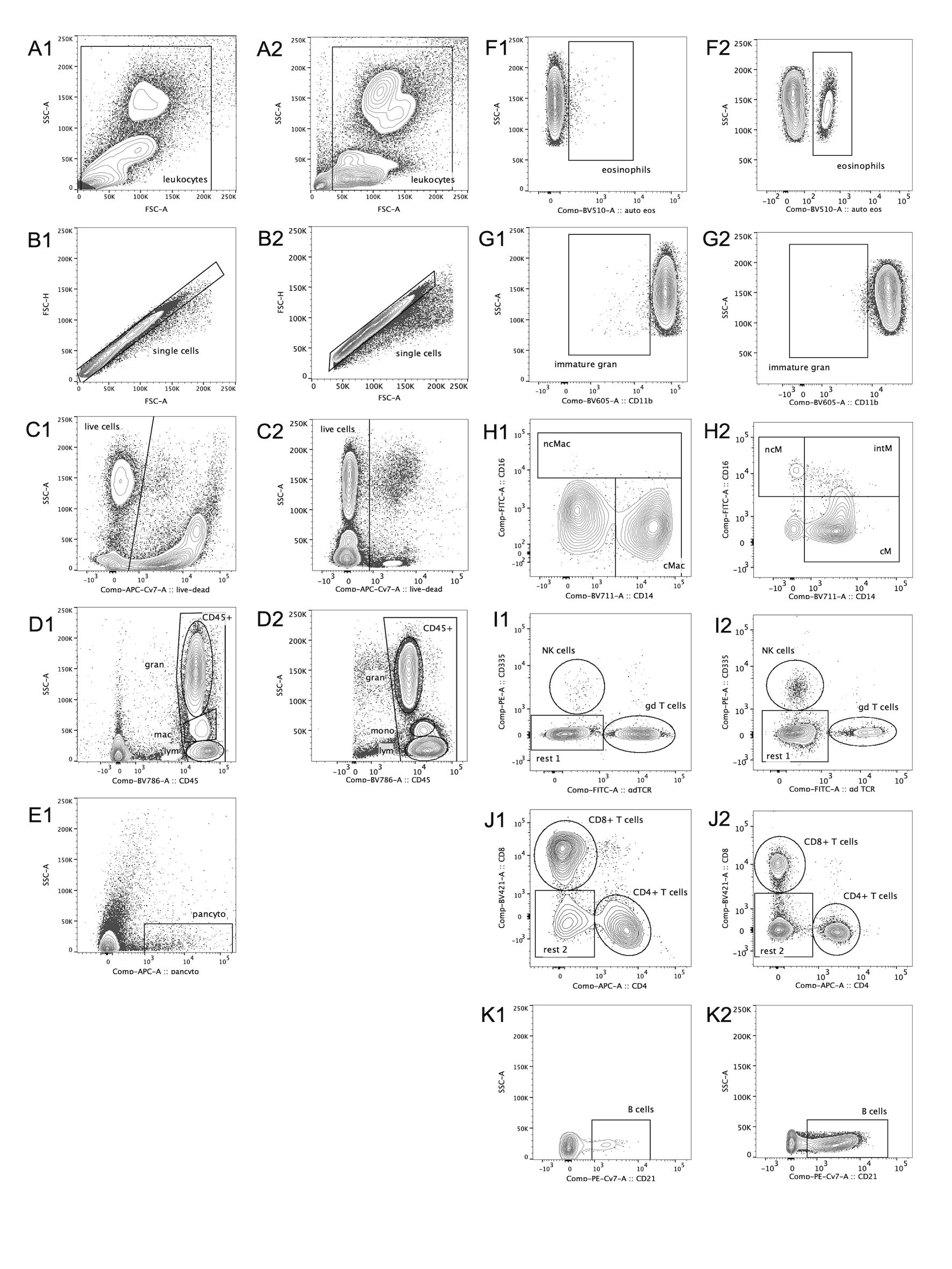

Supplement: Supplementary file 1 [file animals-12-01339-s001.zip › S1_Fig_Gating_strategy.tiff]

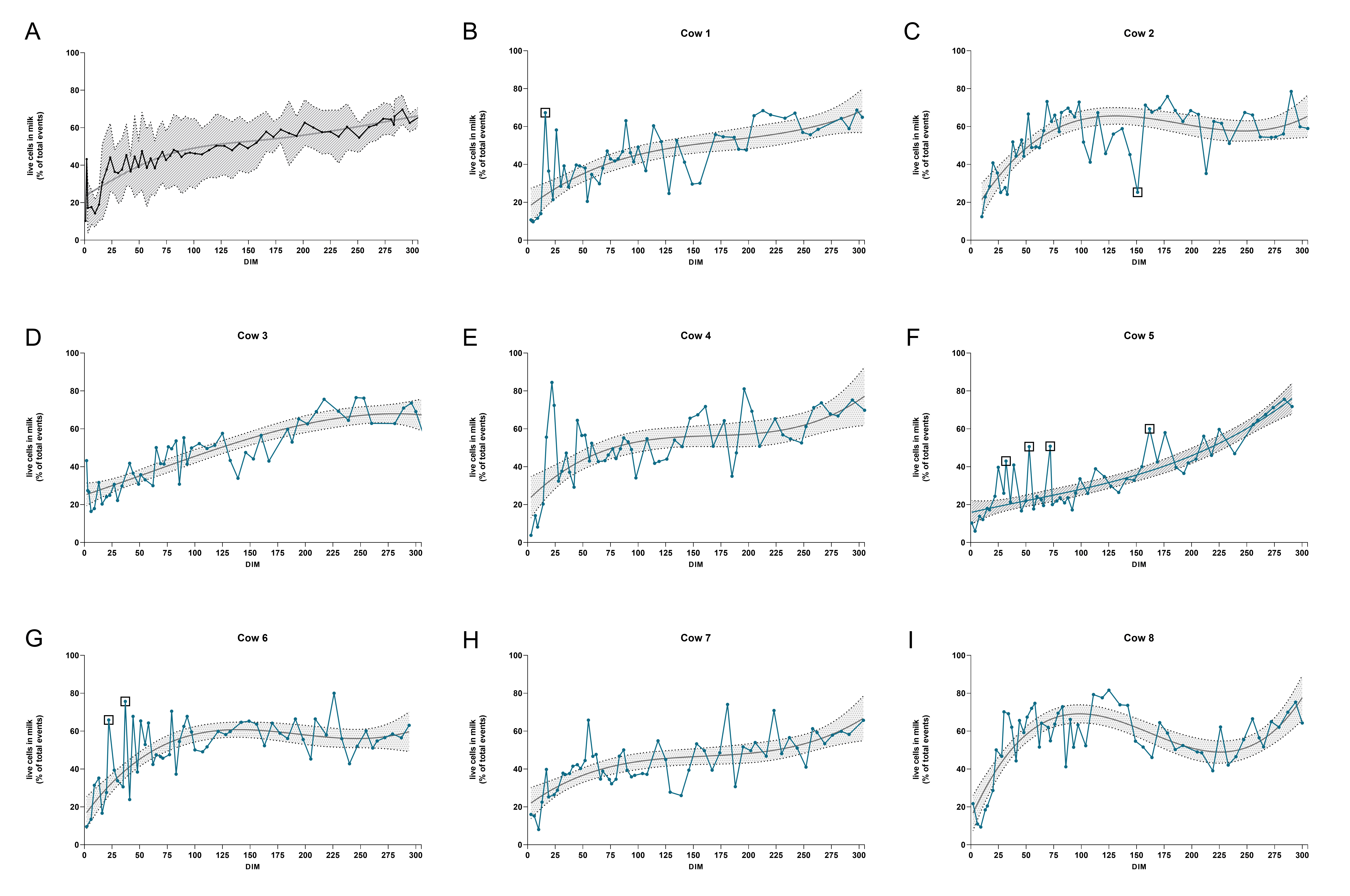

Supplement: Supplementary file 1 [file animals-12-01339-s001.zip › S2_Fig_live_cells_in_milk.tiff]

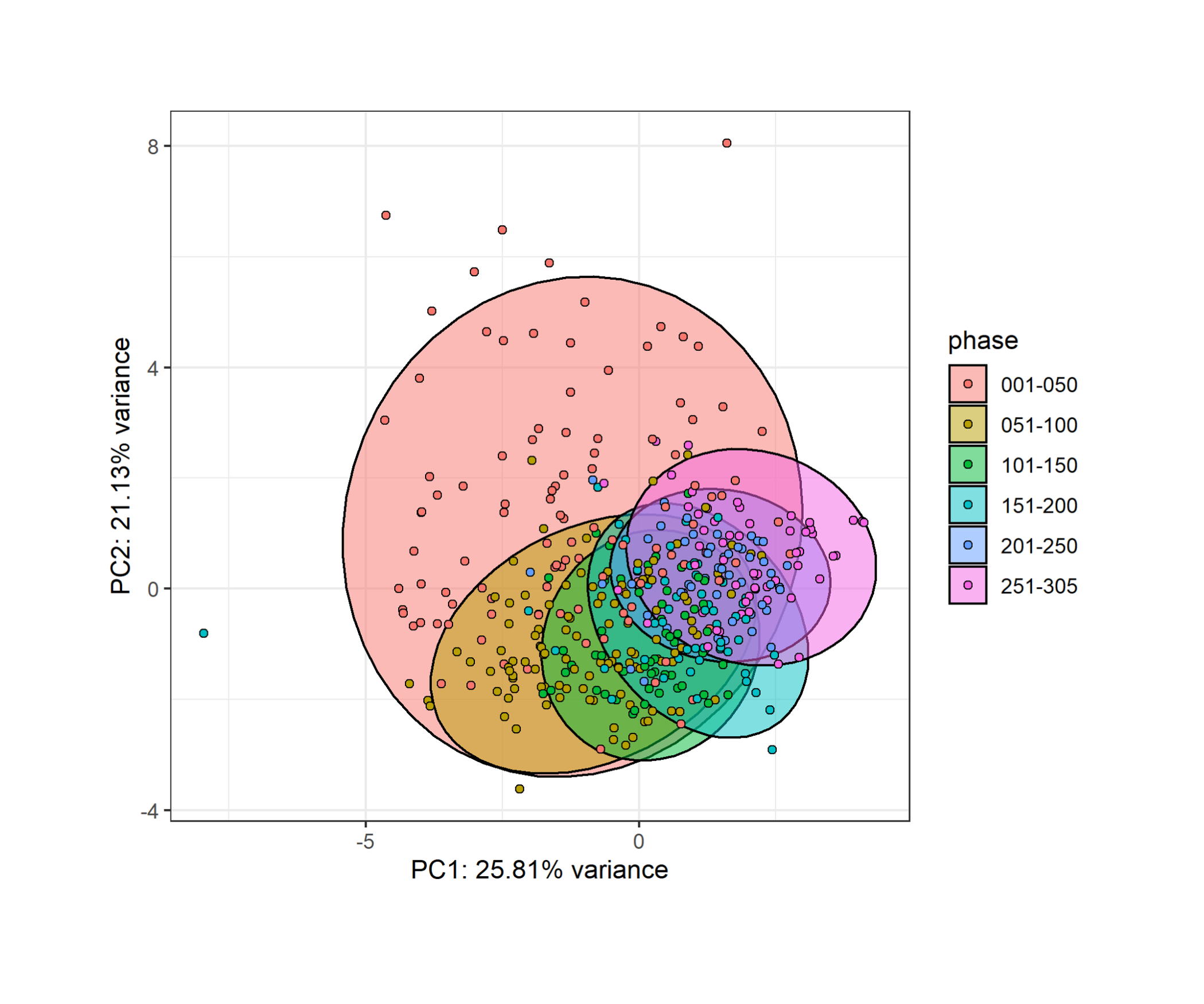

Supplement: Supplementary file 1 [file animals-12-01339-s001.zip › S38_Fig_results_PCA_milk.tiff]

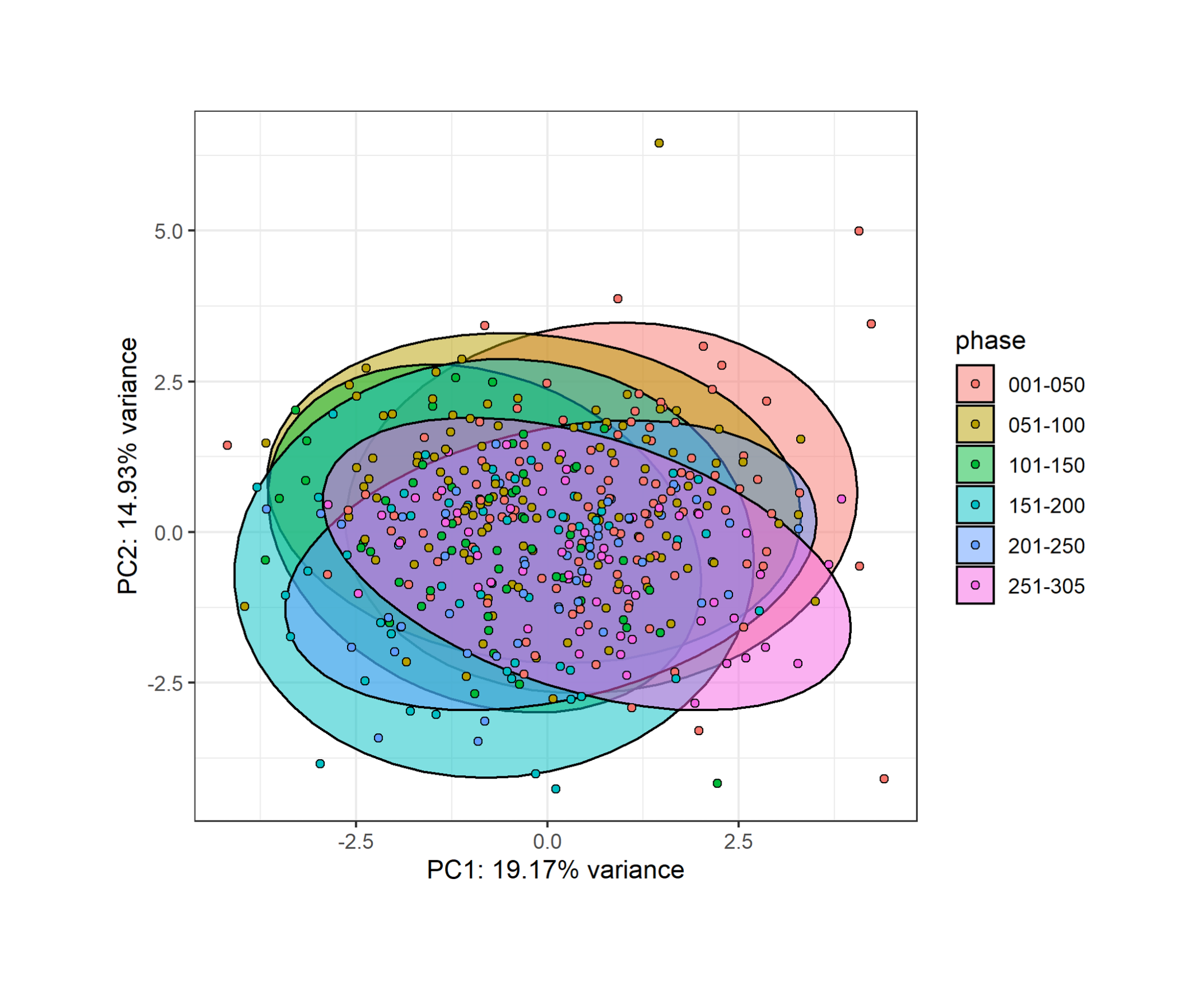

Supplement: Supplementary file 1 [file animals-12-01339-s001.zip › S39_Fig_results_PCA_blood.tiff]

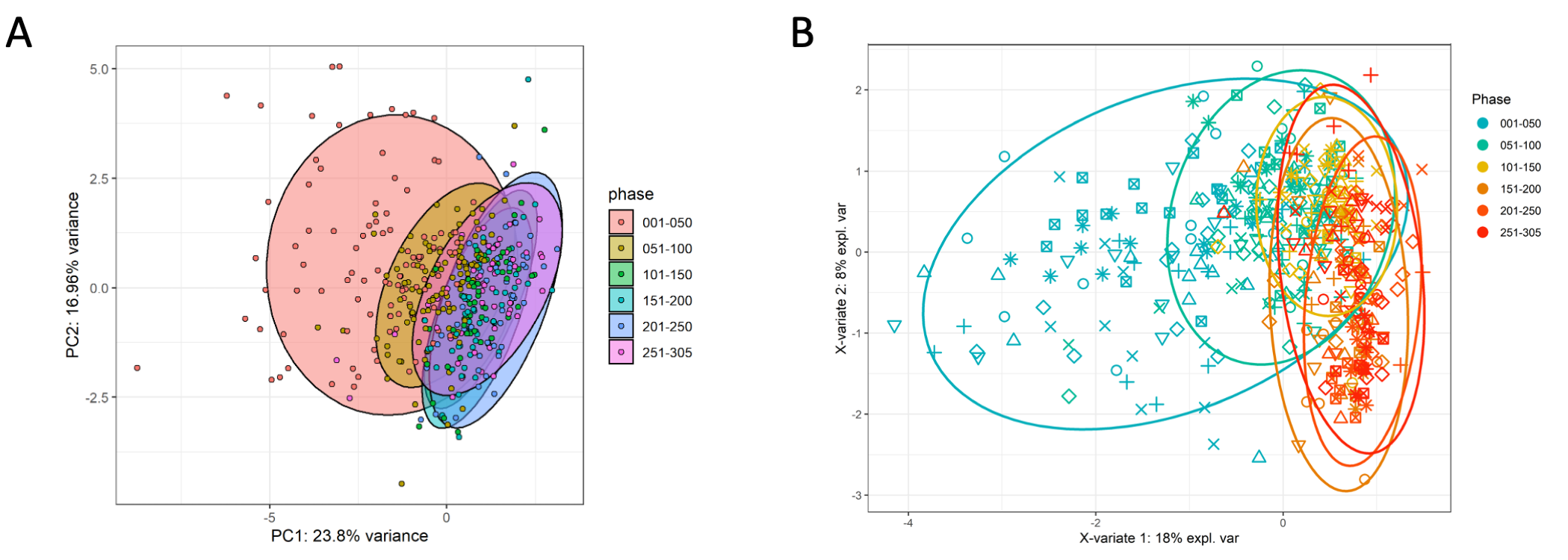

Supplement: Supplementary file 1 [file animals-12-01339-s001.zip › S40_Fig_results_PCA,_sPLSDA_external_lab.tiff]

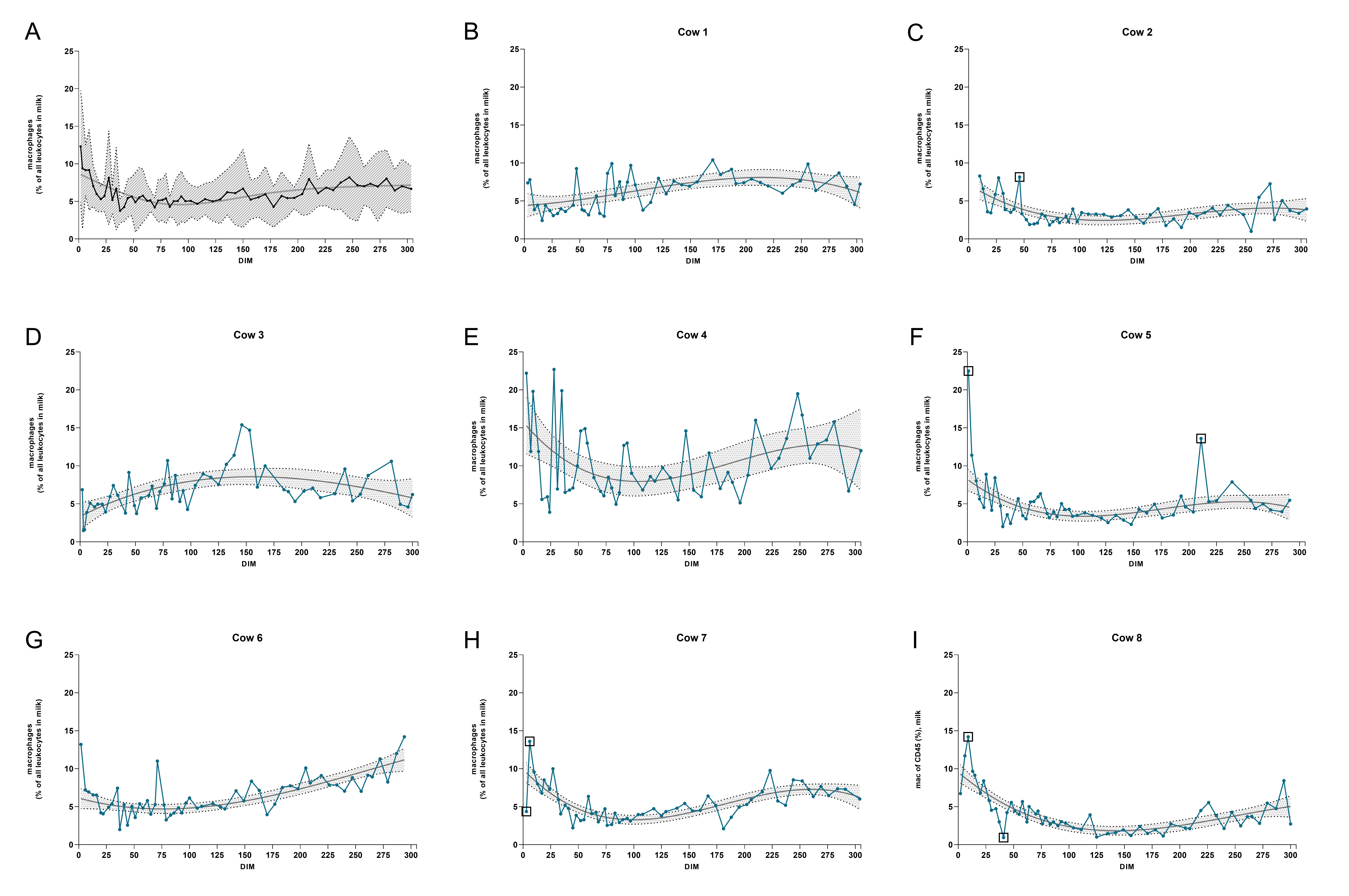

Supplement: Supplementary file 1 [file animals-12-01339-s001.zip › S6_Fig_macrophages_in_milk.tif]
